# Automated Calibration for Rapid Optical Spectroscopy Sensor Development for Online Monitoring

**DOI:** 10.1021/acssensors.4c02211

**Published:** 2024-09-19

**Authors:** Hunter B. Andrews, Luke R. Sadergaski

**Affiliations:** Radioisotope Science and Technology Division, Oak Ridge National Laboratory, 1 Bethel Valley Rd., Oak Ridge, Tennessee 37830, United States

**Keywords:** automation, spectrophotometry, online monitoring, lanthanides, calibration, transient learning

## Abstract

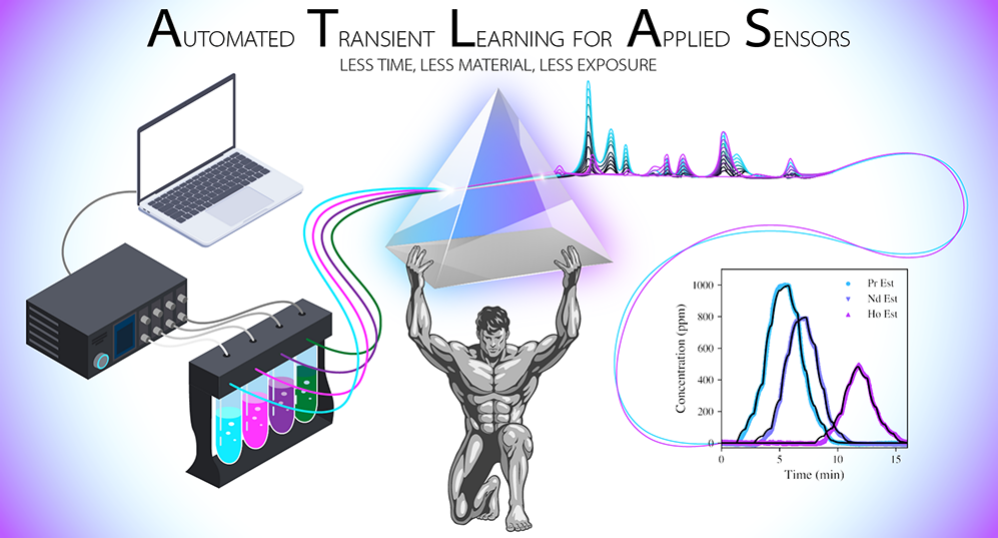

An automated platform has been developed to assist researchers in the rapid development of optical spectroscopy sensors to quantify species from spectral data. This platform performs calibration and validation measurements simultaneously. Real-time, in situ monitoring of complex systems through optical spectroscopy has been shown to be a useful tool; however, building calibration models requires development time, which can be a limiting factor in the case of radiological or otherwise hazardous systems. While calibration time can be reduced through optimized design of experiments, this study approached the challenge differently through automation. The ATLAS (Automated Transient Learning for Applied Sensors) platform used pneumatic control of stock solutions to cycle flow profiles through desired calibration concentrations for multivariate model construction. Additionally, the transients between desired concentrations based on flow calculations were used as validation measurements to understand model predictive capabilities. This automated approach yielded an incredible 76% reduction in model development time and a 60% reduction in sample volume versus estimated manual sample preparation and static measurements. The ATLAS system was demonstrated on two systems: a three-lanthanide system with Pr/Nd/Ho representing a use case with significant overlap or interference between analyte signatures and an alternate system containing Pr/Nd/Ni to demonstrate a use case in which broad-band corrosion species signatures interfered with more distinct lanthanide absorbance profiles. Both systems resulted in strong model prediction performance (RMSEP < 9%). Lastly, ATLAS was demonstrated as a tool to simulate process monitoring scenarios (e.g., column separation) in which models can be further optimized to account for day-to-day changes as necessary (e.g., baseline correction). Ultimately, ATLAS offers a vital tool to rapidly screen monitoring methods, investigate sensor fusion, and explore more complex systems (i.e., larger numbers of species).

The use of optical spectroscopy for real-time, in situ monitoring is continuing to grow, particularly in applications involving complex or hazardous environments. Critical examples of this use are in the nuclear industry, where real-time monitoring via optical spectroscopy is being used in applications across the fuel cycle, including enrichment monitoring, advanced reactor designs, and radioisotope production.^1−9^ In this field, remote monitoring provides operators insight into the processes and systems being used that otherwise rely on measurements through manual sampling. These manual measurements typically involve turnaround times on the scale of days to weeks because of the need for removal from radiological environments (i.e., hot cells/gloveboxes), dilutions, and the associated analytical time. This requirement results in all analyses becoming holding points that can limit the production throughput. Additionally, the analytical results are always received after a test or process has already been completed, leaving no room for online tuning or corrections.

Remote monitoring based on optical spectroscopy offers process insight nearly immediately. This method is relatively simple to install, using fiber optics to send and receive optical signals. Although simple to set up, calibrating these analytical tools can be a challenge, especially with radioactive materials. Researchers desire to minimize the number of samples needed to train a spectroscopy model, which regresses spectral signatures to provide quantifiable species concentrations, for two reasons. First, many of the chemical systems that are under investigation involve resource-limited materials that prohibit a superfluous calibration set. Second, because these samples may be radioactive, they pose an exposure risk to the workers, who are prepping and testing samples. Previous work from our group has focused on using optimal design of experiments to reduce the number of calibration samples significantly.^10−13^ These studies have demonstrated equitable models constructed using only 20 samples from an optimal design as opposed to 125 samples (i.e., 5 levels, 3 species, 5^3^ = 125) from a traditional full factorial design. Additionally, optimized calibration transfer methods to mitigate the need for retraining models day-to-day or instrument-to-instrument have been explored along with automated preprocessing and feature selection for model optimization.^14−17^ Although these studies have progressed in the ability to construct multivariate regression models efficiently, automating sample preparation, spectral acquisition, and model construction is the inherent next evolution of the model building approach.

This study presents the development of an automated calibration system for optical spectroscopy models. This system was demonstrated by using spectrophotometry, or absorbance spectroscopy, on multiple analytical systems, illustrating its flexibility for many project needs. Although absorbance spectroscopy traditionally uses extinction coefficients for direct concentration analysis (i.e., Beer’s law), the univariate relation between absorbance and concentration breaks down in complex systems in which bands overlap or species interact with one another, which is the case in many radioisotope separation processes. In this work, well-established partial least-squares regression (PLSR) models were evaluated.

This article highlights three major points: (1) the development of an automated pumping system to train optical models using only stock solutions, (2) a corresponding program to extract spectra from the measured transients from the automated calibration run and constructed model, and (3) the demonstration of this system to calibrate absorbance spectroscopy models for two analytical systems as well as a demonstration of how the same system can be used to test models in simulated process runs. This automated system has been named ATLAS: Automated Transient Learning for Applied Sensors. Despite only testing nonradioactive surrogates, the automated calibration system is constructed such that it is amenable to radioactive samples, can be installed in gloveboxes, or can be extended to other fields where online monitoring is needed (e.g., pharmaceutical, petroleum, food, etc.). The analytical systems studied include Pr(III), Nd(III), and Ho(III) and Pr(III), Nd(III), and Ni(II). Here, the first system was representative of the chemical separations between lanthanides and actinides. These systems are complicated because of the overlapping absorbance bands of the lanthanides. The second system was selected to demonstrate the rapid “plug and play” capabilities of the system by quickly swapping one analyte. Additionally, Ni(II) is a corrosion product with broad absorbance features, providing a different challenge for an optical model. This state-of-the-art approach can be leveraged for rapid and robust online monitoring applications to support various applications such as the production of ^147^Pm and medical isotopes like ^166^Ho, as well as other chemical systems beyond the nuclear field.

## Experimental Section

### Materials

All chemicals were commercially obtained (American Chemical Society grade) and used as received, unless otherwise stated. Certified inductively coupled plasma optical emission spectroscopy 10,000 ppm Pr(III), Nd(III), Ho(III), and Ni(II) standards were purchased from High-Purity Standards. Concentrated HNO_3_ (70%) was purchased from Sigma-Aldrich. Stock solutions were diluted to 3000 ppm using deionized (DI) water (18.2 MΩ cm^–1^ at 25 °C). During automated runs, stock solutions were diluted using 2% HNO_3_ prepared from concentrated HNO_3_ and DI water.

### Instrumentation and Equipment

**Figure 1:**
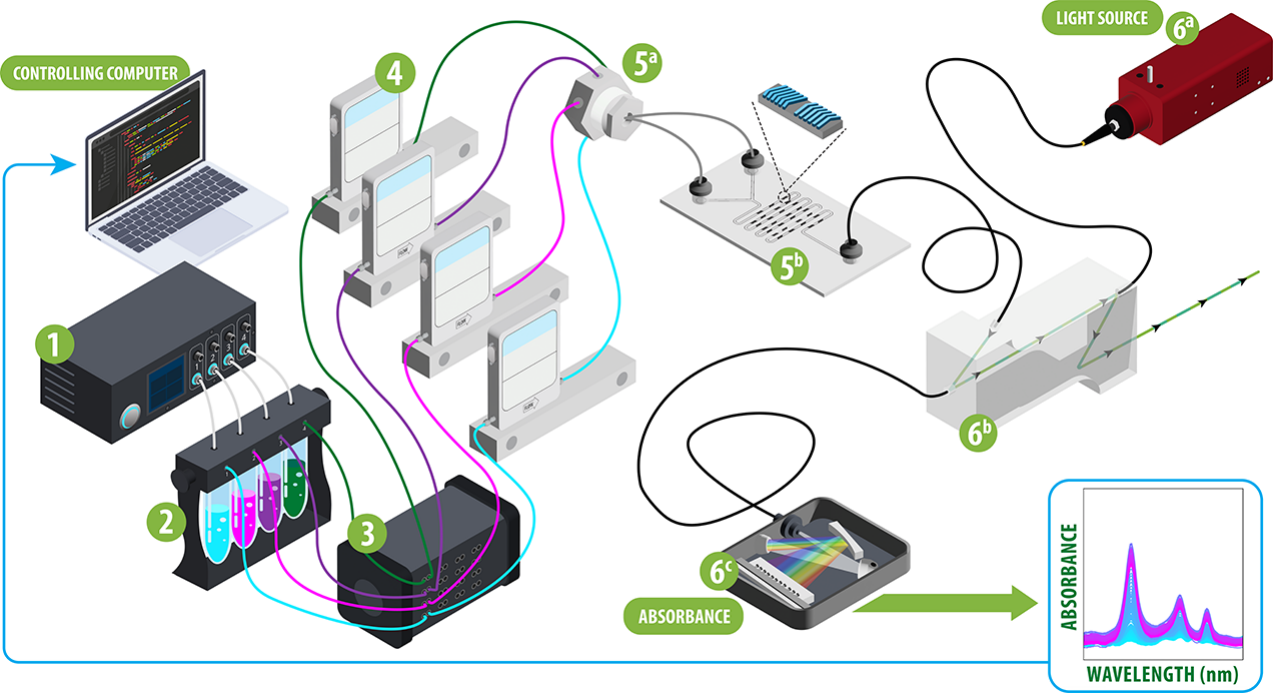
Notional schematic of the ATLAS system with the following components: (1) a pneumatic flow controller, (2) sample reservoirs, (3) an electron valve matrix, (4) flow meters, (5a) line combiner, (5b) herringbone mixer chip, (6a) broadband light source, (6b) Z flow cell, and (6c) UV–vis spectrometer. Note that component 6 may be replaced with alternate or additional sensors.

The automated calibration system, ATLAS, was constructed using a pneumatic OB1 flow control system (Elveflow) connected to four sample reservoirs configured with in-line MKS flow meters (Bronkhurst) on each flow channel to create proportional–integral–derivative (PID) controllers for precise flow control. A MUX flow switch matrix (Elveflow) was installed between the sample reservoirs and the flow meters to provide electronically controlled valves to rapidly stop the flow and prevent reverse flow. The fluid lines at the output of the flow meters were then connected into a single line, which was then passed through a microfluidic herringbone mixer chip (Darwin Microfluidic) to ensure that solutions were mixed thoroughly before measurement. The mixed sample stream then was passed through a 50 mm PEEK Z flow cell (Ocean Insight). Absorbance measurements were made using a W halogen lamp (Thorlabs) and a QEPro ultraviolet–visible (UV–vis) spectrometer (Ocean Insight). Multimode, low-OH fibers with a 400 μm core diameter and a length of 2 m (Thorlabs) were used on the excitation side. The collection side used 2-to-1 600 μm core diameter ZBLAN fibers (Thorlabs) to allow both UV–vis and NIR spectrometers to be used with ATLAS simultaneously. All spectra were collected in 500 ms increments, with each being the average of 10 replicates collected with a 40 ms exposure time. The output of the flow cell was then sent to a waste collection vial. A notional schematic of ATLAS is shown in Figure 1.

A generic D-optimal design for three analytes was built with Design-Expert (v.11.0.5.0, Stat-Ease Inc.) within the Unscrambler software package (Camo Analytics). This design included 10 model samples and 10 lack-of-fit samples to cover an adequate amount of the fractional design space (>99%) (see Table S1). An in-house Python script was developed to control the liquid handling in ATLAS, including the PID control of the pneumatic pumps, check values, and conversion of the calibration sample matrix into a flow plan. The control script outputs the pressure and flow record of each analyte to an Excel file for later model use. In addition to using ATLAS for calibration, process monitoring was simulated (e.g., column effluent) by generating new flow plans, as desired.

### Chemometric Modeling

A second Python script was built to import the flow record output of the control script and then the spectral data. The script was built with functionality for the user to toggle the truncation of spectra to a desired wavelength range, smooth the data as desired with Savitzky–Golay filters, or do baseline correction at a given wavelength or at the minimal absorbance value in each spectrum. The user may then select wavelengths to monitor for an initial comparison between the absorbance signals and the flow record.

The user must enter the time duration for each flow step (as configured in the control program) and the number of spectra to extract at each step for the calibration (e.g., 20 spectra). The script then produces a plot comparing the flow record to the univariate absorbance response over time. The selected spectra are indicated by marked points to allow the user to ensure that the system has equilibrated from the previous flow settings before the spectra are extracted (see Figure S1). The extracted spectra are then used to build a calibration library. The time stamps of the extracted spectra are used along with the flow record to generate the concentration matrix. Next, both the spectra and concentration values are averaged such that one value exists for each flow step, providing 20 samples and two blanks from the beginning and end of the autocalibration run.

Chemometric modeling was completed using the SciKit Learn library in Python.^18^ Partial least-squares regression (PLSR) is a widely used multivariate modeling technique for analyzing data when the dimensionality of the predictors (i.e., spectra) far exceeds that of the number of samples, as well as when data sets have issues associated with multicollinearity.^19−22^ This issue is commonly the situation with optical spectroscopy, and PLSR is well suited for predictive models under these circumstances.^23,24^ PLSR models were built using the calibration library extracted from the run. Cross-validation (CV) was performed using both leave-one-out cross-validation and *k*-fold (*k* = 5) methods. A leave-one-out approach iteratively builds models with a single sample left out and evaluates the model using the sample left out. A k-fold approach splits the data into k splits and evaluates the model built using k-1 splits using the samples left out. Latent variables (LVs) for PLSR models were determined by visual inspection of a root-mean-square error (RMSE) of CV (RMSECV) versus LV included in the model. RMSE is a measure of prediction performance and is defined as
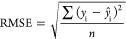
1where *y*_i_ is the known concentration,  is the model-predicted concentration, and *n* is the number of samples. RMSE can be calculated for calibration (RMSEC) when all predicted samples are in the training set; RMSEC is a measure of the predictive spread of the calibration model. RMSE can also be calculated during various CV schemes (RMSECV) or for the prediction of validation sets (RMSEP), which are never used for training. For ease in model comparison, percent RMSEs are reported by scaling RMSE values by the median of the design space concentration (e.g., 500 ppm if the design ranges from 0 to 1000 ppm). When building PLSR models, the last LV to provide a significant reduction in the RMSECV for each analyte was selected. While lower RMSE values are desired regardless of the type, it is typical for RMSEP values to be on par or greater than the RMSECV values for the same model. Ultimately, RMSEP values provide the best metric for understanding a model’s ability to properly predict analyte concentration. A pseudounivariate approach was used to calculate the limits of detection (LODs) based on how well the model predicts the samples used to construct it.^25^ The LOD served as a measure of the model sensitivity.

## Results and Discussion

### System Design and Optimization

Prior to automated runs, a procedure to prime ATLAS was developed to provide a repeatable operation. The liquid-handling components of the system were stored in wet configurations with all channels containing 2% HNO_3_. This configuration prevented analytes from depositing within the system and causing subsequent cross-contamination. To prime the system, the stock solution vials were exchanged for the selected analytes. Then, each channel was run at a constant flow rate until the absorption spectra stabilized. Next, the 2% HNO_3_ stock was run until the absorption spectra returned to its baseline. This process allowed all plumbing up to stage 5a in Figure 1 to be primed. A similar procedure was run with 2% HNO_3_ for cleaning and storing the system.

Another vital point of consideration for optimizing liquid handling for ATLAS was its ability to repeatably control the fluid flow such that dilutions of stock solutions were consistent. Several mixer options were compared, including simply allowing natural mixing between the line combination (stage 5a) and the flow cell, using an active stir mixer chip with stir bars, or a herringbone mixer chip (stage 5b). Natural mixing was inadequate because of the laminar flow of the small-diameter tubing. The active stir mixer that was provided aided repeatability but required a stir plate and reduced flow rates. The herringbone mixer proved superior because of its throughput and its mixing mechanism being passive.^26^ The repeatability of the system’s mixing is shown in Figure S2.

Mixing time was characterized to determine the time each flow level should be run to effectively reach a steady state such that calibration spectra could properly be extracted. This was determined to be 90 s based on the time for a spectral profile to stabilize as seen in Figure S2. Because spectra were collected during these transients, they also could be leveraged for a validation data set. However, for these spectra to form useful validation sets, the corresponding concentration profiles needed to be computed. Using the principles of plug flow reactors, the concentration profiles at each change of the flow rates were computed using
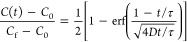
2where *C*(*t*) is the concentration at time *t* after the onset of a flow change, *C*_0_ is the concentration prior to the flow change, *C*_f_ is the expected concentration after the transient, τ is a constant related to residence time, and *D* is a dimensionless dispersion coefficient. Constants *D* and τ were estimated by fitting preliminary transient profiles to be 0.25 and 0.35 s, respectively. Eq 2 was used to solve for the concentration profile, *C*(*t*), for each transient as the flow rates were changed, and the profiles were then appended to one another to form the estimated concentration profile for a given test run. These estimated concentration profiles can be seen overlaid with the transients during the mixing tests used to determine *D* and τ in Figure S2. These transients are similar to those reported by Schwantes et al. when monitoring spectroscopic signatures from feed and raffinate streams during chemical separations on a bank of centrifugal contactors.^27^

### Automated Calibration Demonstration 1

ATLAS was first used to demonstrate calibration for a Pr, Nd, and Ho system—three lanthanides with several absorption bands each, which overlap at varying levels. These characteristic absorption peaks correspond to intra-4f transitions.^10^ This system is a common monitoring scenario area for many nuclear separation systems in which Beer’s law can break down and multivariate models are needed.^9^

**Figure 2:**
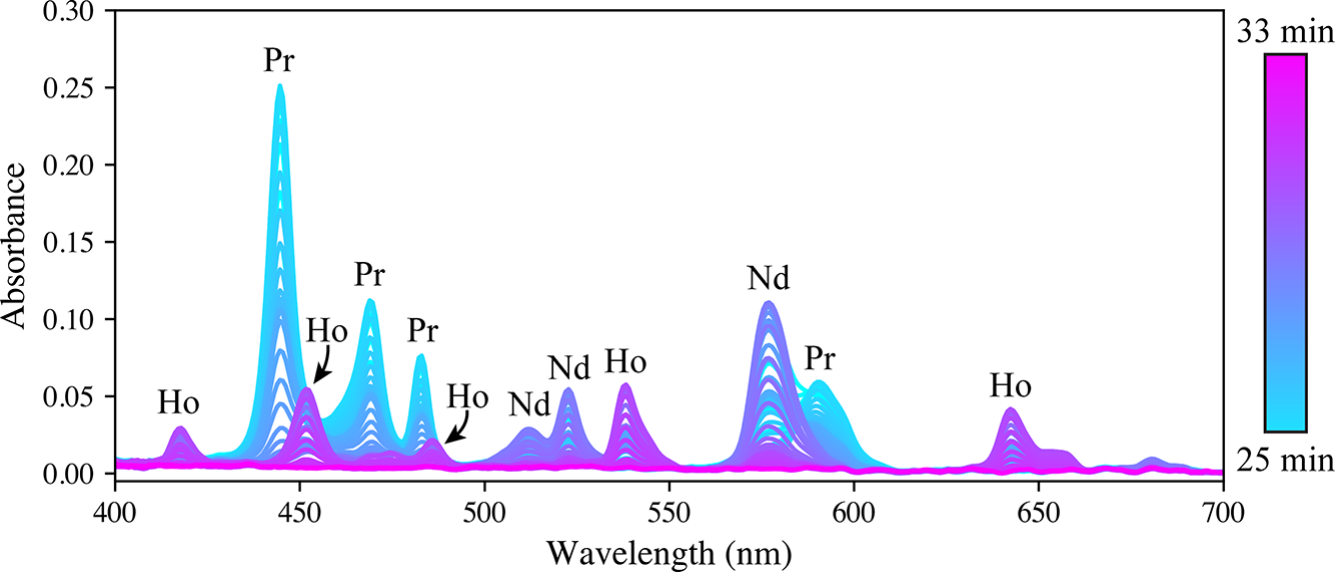
Spectra collected during the last 8 min of the automated calibration run, with Pr, Nd, and Ho peaks labeled. The interferences between Pr and Ho, as well as Pr and Nd, are clearly shown.

The 20 D-optimal sample compositions were cycled through ATLAS with blanks at the beginning and end of the run. The flow record and univariate signal response versus time are shown in Figure S1. Spectra were imported, smoothed using a five-point window, and baseline-corrected based on the minimum absorbance value in each spectrum. The spectra from the last 8 min of the run are shown in Figure 2. This figure highlights the wealth of information collected using ATLAS and displays interference between analyte absorbance bands.

Initially, univariate calibrations were investigated (Figure S3). A linear response was shown for each species, as expected. However, Pr was particularly affected by the presence of other species, as seen in the significant scatter in the univariate response. This scatter is due to the heavily overlapping absorbance region between 400 and 500 nm (Figure S4). Interestingly, each species shows greater variance at the edge of the design space (i.e., 0 and 1000 ppm). Overlapping peaks and divergence from the Beer’s law relation led to the use of multivariate modeling.

**Figure 3:**
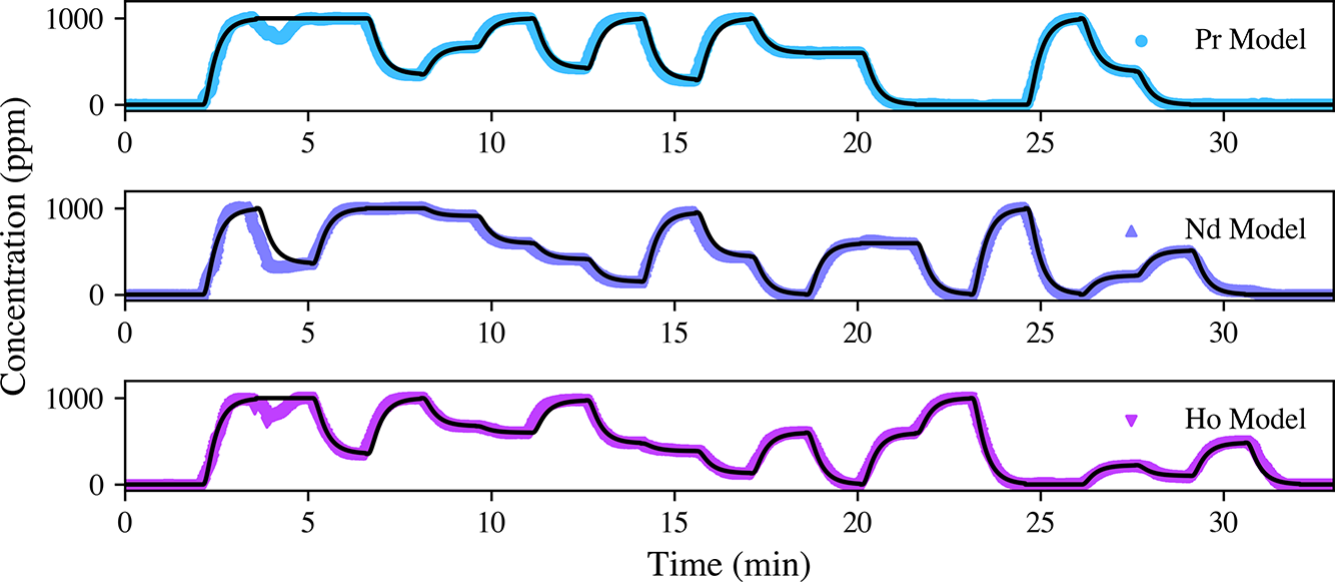
PLSR model-predicted concentration profiles for Pr, Nd, and Ho compared with the expected concentration profiles calculated using eq 2 (black lines). The deviance from the expected profile before 5 min is due to fluctuations in the flow control PID and was omitted from validation metrics.

The ATLAS modeling script was designed to rapidly construct multivariate PLSR models. The script extracts calibration spectra, performs CV for LV selection, and then demonstrates the model prediction capabilities by estimating the concentration profiles of the transients when the flow rates are changed. The prediction metrics for the PLSR model are listed in Table 1. The calibration and CV metrics showed phenomenal performance, and clearly, the issues associated with the univariate responses were overcome. The PLSR model prediction of the transient concentration profiles is shown in Figure 3. Apart from the period prior to 5 min, the model’s predictions align well with the expected profiles. The discrepancy before 5 min is due to the PID flow control being less stable when all channels were first activated. This issue is shown in the flow record in Figure S1 where the DI stock pressure spikes; fortunately, the system always recovered, and this period of the transient profile can be omitted from the RMSEP calculation. The RMSEP falling below 10% indicates satisfactory performance and, if we consider that the expected profiles are estimates themselves, then these models likely fall into the strong performance category.^28^ Additionally, the LODs for the PLSR models are provided in Table 1, highlighting the sensitivity of the models.

**Table 1:** Pr, Nd, and Ho Prediction Metrics for ATLAS Multivariate Models

species	RMSEC (%)	RMSECV (%)	RMSEP (%)	*R*^2^ calibration	*R*^2^ CV	*R*^2^ Prediction	LOD (ppm)
Pr	0.99	1.2	8.7	0.9999	0.9995	0.9853	17
Nd	2.3	1.9	5.5	0.9991	0.9966	0.9935	40
Ho	1.2	1.5	5.8	0.9997	0.9989	0.9915	22

*PLSR models were built with LV = 3 (Pr), 2 (Nd), and 3 (Ho). *k*-fold CV was performed. RMSEP *n* = 3211.

### Automated Calibration Demonstration 2

To demonstrate the flexibility of ATLAS, a second calibration was performed on a separate system. Previously, the Pr, Nd, and Ho system provided a use case in which species’ absorbance bands overlapped, causing trouble with traditional univariate models. In the second calibration, Ho(III) was replaced with Ni(II). The Pr, Nd, and Ni system examined another use case in which lanthanides, which have more narrow absorbance bands, are interfered with by species with very broad absorbance profiles such as the transition metal Ni.

**Figure 4:**
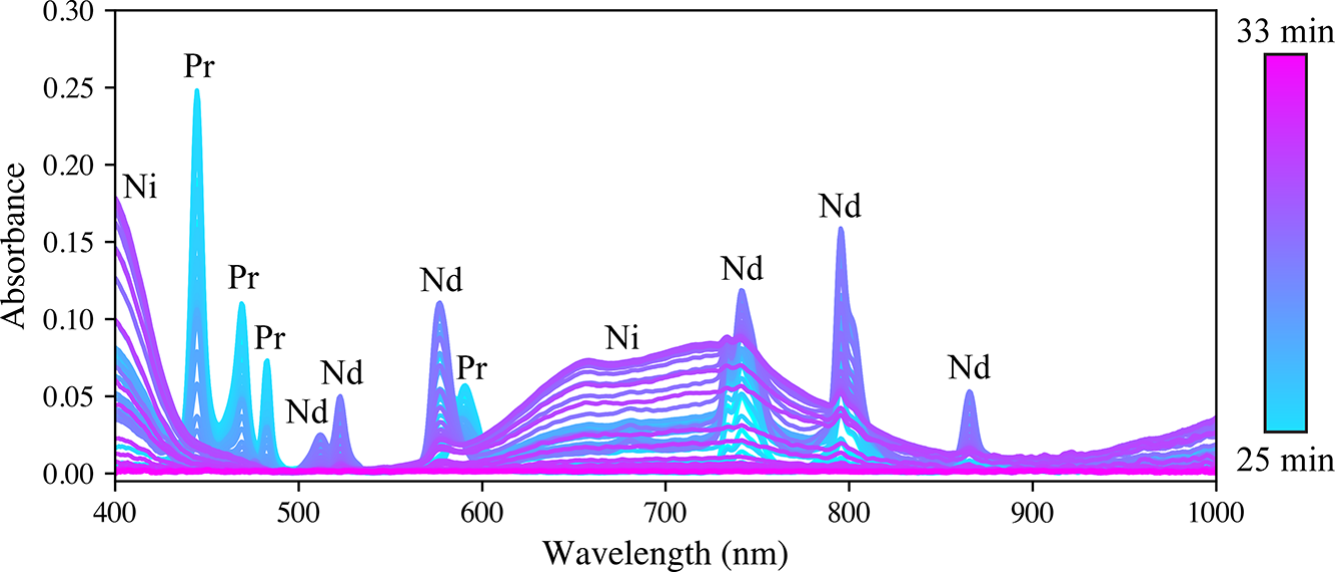
Spectra collected during part of the automated calibration run with Pr, Nd, and Ni. The broad features from 400 to 450 nm and 600 to 850 nm correspond to Ni.

For ATLAS, this system was rapidly calibrated by switching the stock solution, priming the third channel, and then running the automated procedure. The collected spectra are shown in Figure 4. PLSR models were constructed with the same procedure discussed previously, and the resultant models were used to predict the transient concentration profiles (Figure S5). This work highlights the utility of ATLAS to rapidly develop models for monitoring needs as they arise. The PLSR model prediction metrics are provided in Table 2. Similar prediction performance to that of the Pr, Nd, and Ho calibrations was achieved. Interestingly, the LODs for Pr and Nd were slightly increased in the presence of Ni, which can be attributed to its broad absorbance band interference. Fortunately, the models were sufficiently sensitive to monitoring the ATLAS transients.

**Table 2:** Pr, Nd, and Ni Prediction Metrics for ATLAS Multivariate Models

species	RMSEC (%)	RMSECV (%)	RMSEP (%)	*R*^2^ calibration	*R*^2^ CV	*R*^2^ prediction	LOD (ppm)
Pr	1.8	2.0	8.4	0.9995	0.9986	0.9866	32
Nd	3.3	4.2	7.6	0.9981	0.9945	0.9874	58
Ni	1.8	2.1	5.7	0.9994	0.9972	0.9917	31

*PLSR models were built with LV = 3 (Pr), 2 (Nd), and 2 (Ni). *k*-fold CV was performed. RMSEP *n* = 3213.

Each ATLAS calibration run took 33 min, which represents a tremendous increase in time efficiency compared to manually preparing and collecting spectra for each sample. Based on experience, the time and resource savings for ATLAS can be benchmarked.^13^ Assuming that an ideal worker might prepare a sample gravimetrically by hand at a rate of 4 min for one sample, with 20 samples per D-optimal design and a measurement time of 3 min for each sample, their time would equate to 140 min^13^ The ATLAS runs’ duration represents a 76% reduction in calibration time, and that does not consider the time to manually process data and construct a model. Similarly, the ATLAS run consumed roughly 4 mL of the 3000 ppm of stock solution for each analyte. For comparison, if 20 static calibration samples were prepared (3 mL each), then approximately 10 mL of the stock solutions would be consumed for each analyte.^13^ This equates to ATLAS offering a 60% reduction in sample volume used during the calibration while providing a wealth of information during the transients that cannot be captured with traditional sample preparation. Additionally, faster flow rates, which may be achieved through further flow resistance reduction, could reduce the amount of stock solution required for ATLAS calibrations. Through the reduction in calibration time and volume versus a manual static calibration, ATLAS poses a significant reduction in exposure time for workers when radiological materials are considered. Furthermore, the automated operation of ATLAS leaves the system amenable to future autonomous iterations where machine learning can be used to further refine models by measuring additional compositions and/or optimizing chemometric model hyperparameters.^29−32^

### Simulated Column Demonstration

Although ATLAS was developed primarily for calibration, the ability to program concentration profiles lends itself to also be a valuable tool for simulating monitoring scenarios. Once calibrations are established, they can readily be transferred to flow cells attached to columns and other configurations. A common use case for optical spectroscopy would be to monitor the effluent during column chromatography separations for radioisotope production. Here, the Pr, Nd, and Ho system is quite relevant because column separations of adjacent lanthanides are a common scenario. Optical spectroscopy is a useful tool for scientists and operators to monitor the column effluent and decide when to switch between collection and waste to capture the species of value.^33^

**Figure 5:**
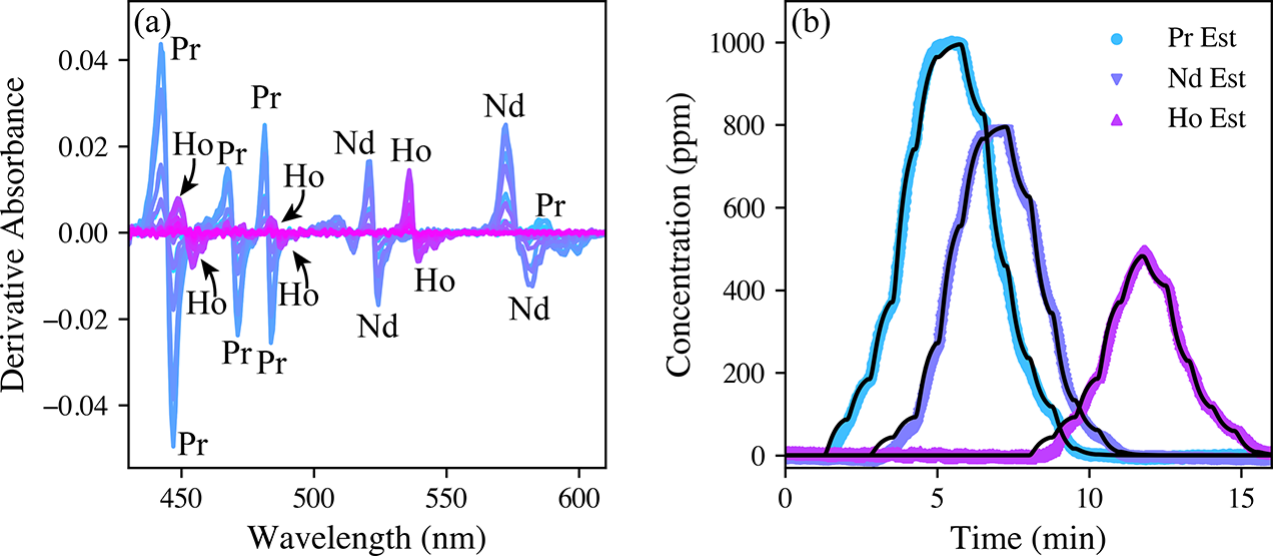
Spectra collected during the ATLAS-simulated column separation after a first-derivative baseline correction (a) and model-predicted concentrations for the Pr, Nd, and Ho test (b). The expected concentration profiles calculated using eq 2 (black lines).

In this simulation, ATLAS was used to simulate a generic column separation of Pr, Nd, and Ho. The test was set up such that Pr and Nd would elute at similar times, whereas Ho would pass through the column at a delayed time.^34^ The ATLAS-generated PLSR models were used to predict the concentration profiles during the simulated column run. This simulation served as an additional benefit of testing the ATLAS calibration on data collected at a different time and date. This work found that because of slight variations in the absorbance spectra, the standard baseline correction procedure used previously was not sufficient. Rather, the PLSR model was reconstructed in ATLAS using a first-derivative Savitzky–Golay filter to handle baseline shifts (Figure 5a). This model demonstrated predictive metrics similar to those of the previous ATLAS calibration (Table S2), but this model was more capable of monitoring the simulated test. The model-predicted concentration profile for the simulated test is shown in Figure 5b. For reference, the concentration profile predicted using the PLSR model without derivative baseline correction is shown in Figure S6. Future iterations of simulated tests using ATLAS could be designed with more intent to represent the behavior of specific column types (e.g., resin type, gravity- or pressure-driven, etc.).

## Summary and Conclusions

As optical spectroscopy continues to grow as a vital tool for real-time monitoring of chemical processes in complex environments, the need to rapidly calibrate models for these applications grows as well. Previous work has demonstrated the ability to reduce resource utilization when building models through optimal design of experiments; however, this study has demonstrated a substantial leap in improving model development turnaround time.

The ATLAS system automatically calibrates a multivariate model for three species in as little as 33 min, which is a 76% reduction in time from that of optimized human calibrations. ATLAS uses pneumatic pumps and concentrated stock solutions and can be operated with minimal user interaction and minimal sample usage (60% less than manual calibrations). These capabilities not only make ATLAS ideal for meeting the many demands for optical spectroscopy monitoring models but also make it amenable to systems with radioactive species. The pressure-driven pumps, light sources, spectrometers, and operating computer can be stationed outside of a radiological hood or glovebox, and because only the stock solutions need to be prepared by the operator, the radiological dose would be significantly reduced for the user compared with traditional approaches. However, it is important to acknowledge that inspection by experienced researchers would still be needed rather than blindly trusting an automated system to minimize false predictions.

Lastly, although ATLAS has been demonstrated here for absorbance spectroscopy, it can easily be reconfigured for other optical sensors (e.g., Raman and laser-induced fluorescence) or radiometric sensors (e.g., alpha and gamma spectroscopy). Future work will explore extending ATLAS to these alternative measurement modalities, increasing the throughput of the system to reduce resources further, exploring autonomous operation using machine learning, and increasing the number of species in each calibration. Many studies have been limited to three species because of the burden of sample preparation for larger systems; ATLAS opens the door to exploring more realistic systems by extending the number of channels and only requiring the user to prepare a few additional stock solutions.

## Supporting Information Available

Univariate calibration curves, mixing curves, spectra, design of experiments, and model metrics (PDF)

## References

[ref1] Chan G. C.-Y.; Mao X.; Martin L. R.; Trowbridge L. D.; Russo R. E. Direct uranium enrichment assay in gaseous uranium hexafluoride with laser induced breakdown spectroscopy. J. Radioanal. Nucl. Chem. 2022, 331 (3), 1409–1421. 10.1007/s10967-022-08215-2.

[ref2] Andrews H. B.; McFarlane J.; Myhre K. G. Monitoring Noble Gases (Xe and Kr) and Aerosols (Cs and Rb) in a Molten Salt Reactor Surrogate Off-Gas Stream Using Laser-Induced Breakdown Spectroscopy (LIBS). Appl. Spectrosc. 2022, 76, 988–997. 10.1177/00037028221088625. 35537200

[ref3] Felmy H. M.; Clifford A. J.; Medina A. S.; Cox R. M.; Wilson J. M.; Lines A. M.; Bryan S. A. On-line monitoring of gas-phase molecular iodine using Raman and fluorescence spectroscopy paired with chemometric analysis. Environ. Sci. Technol. 2021, 55 (6), 3898–3908. 10.1021/acs.est.0c06137. 33411509

[ref4] Felmy H. M.; Cox R. M.; Espley A. F.; Campbell E. L.; Kersten B. R.; Lackey H. E.; Branch S. D.; Bryan S. A.; Lines A. M. Quantification of Hydrogen Isotopes Utilizing Raman Spectroscopy Paired with Chemometric Analysis for Application across Multiple Systems. Anal. Chem. 2024, 96, 7220–7230. 10.1021/acs.analchem.4c00802. 38656924

[ref5] Sadergaski L.; Myhre K.; Delmau L. H.; Benker D.; DePaoli D.; Wham R. Spectroscopic and Multivariate Analysis Development in Support of the Plutonium-238 Supply Program; Oak Ridge National Lab.(ORNL): Oak Ridge, TN (United States), 2020.

[ref6] Lascola R.; O’Rourke P. E.; Immel D. M. Development of a Nuclear Fuel Dissolution Monitor Based on Raman Spectroscopy. Sensors 2024, 24 (2), 607 10.3390/s24020607. 38257699 PMC10819358

[ref7] Lines A. M.; Barpaga D.; Zheng R. F.; Collett J. R.; Heldebrant D. J.; Bryan S. A. In Situ Raman Methodology for Online Analysis of CO2 and H2O Loadings in a Water-Lean Solvent for CO2 Capture. Anal. Chem. 2023, 95 (42), 15566–15576. 10.1021/acs.analchem.3c02281. 37787757

[ref8] Lines A. M.; Tse P.; Felmy H. M.; Wilson J. M.; Shafer J.; Denslow K. M.; Still A. N.; King C.; Bryan S. A. Online, Real-Time Analysis of Highly Complex Processing Streams: Quantification of Analytes in Hanford Tank Sample. Ind. Eng. Chem. Res. 2019, 58 (47), 21194–21200. 10.1021/acs.iecr.9b03636.

[ref9] Bryan S. A.; Levitskaia T. G.; Johnsen A. M.; Orton C. R.; Peterson J. M. Spectroscopic monitoring of spent nuclear fuel reprocessing streams: an evaluation of spent fuel solutions via Raman, visible, and near-infrared spectroscopy. Radiochim. Acta 2011, 99 (9), 563–572. 10.1524/ract.2011.1865.

[ref10] Sadergaski L. R.; Toney G. K.; Delmau L. H.; Myhre K. G. Chemometrics and experimental design for the quantification of nitrate salts in nitric acid: Near-infrared spectroscopy absorption analysis. Appl. Spectrosc. 2021, 75 (9), 1155–1167. 10.1177/0003702820987281. 33393348

[ref11] Sadergaski L. R.; Hager T. J.; Andrews H. B. Design of experiments, chemometrics, and Raman spectroscopy for the quantification of hydroxylammonium, nitrate, and nitric acid. ACS Omega 2022, 7 (8), 7287–7296. 10.1021/acsomega.1c07111. 35252718 PMC8892473

[ref12] Sadergaski L. R.; Andrews H. B.; Wilson B. A. Comparing Sensor Fusion and Multimodal Chemometric Models for Monitoring U(VI) in Complex Environments Representative of Irradiated Nuclear Fuel. Anal. Chem. 2024, 96, 1759–1766. 10.1021/acs.analchem.3c04911. 38227702

[ref13] Sadergaski L. R.; Andrews H. B.; Rai D.; Anagnostopoulos V. A. Comparing Designed Training Sets to Optimize Multivariate Regression Models for Pr, Nd, and Nitric Acid Using Spectrophotometry. Appl. Spectrosc. Pract. 2024, 2( (1), ). 10.1177/27551857241243083.

[ref14] Andrews H.; Moon J.; Sadergaski L. R. Leveraging Calibration Transfer Techniques for Remote Monitoring of Samarium and Europium in LiCl Using Laser-Induced Fluorescence Spectroscopy for Radioisotope Production Applications. Ind. Eng. Chem. Res. 2024, 63 (25), 11082–11089. 10.1021/acs.iecr.4c01148.

[ref15] Mishra P.; Passos D. Deep calibration transfer: transferring deep learning models between infrared spectroscopy instruments. Infrared Phys. Technol. 2021, 117, 103863 10.1016/j.infrared.2021.103863.

[ref16] Workman J. J. A review of calibration transfer practices and instrument differences in spectroscopy. Appl. Spectrosc. 2018, 72 (3), 340–365. 10.1177/0003702817736064. 28929781

[ref17] Andrews H. B.; Sadergaski L. R.; Cary S. K. Pursuit of the Ultimate Regression Model for Samarium (III), Europium (III), and LiCl Using Laser-Induced Fluorescence, Design of Experiments, and a Genetic Algorithm for Feature Selection. ACS Omega 2023, 8, 2281–2290. 10.1021/acsomega.2c06610. 36687031 PMC9850777

[ref18] Pedregosa F.; Varoquaux G.; Gramfort A.; Michel V.; Thirion B.; Grisel O.; Blondel M.; Prettenhofer P.; Weiss R.; Dubourg V. Scikit-learn: Machine learning in Python. J. Mach. Learn. Res. 2011, 12, 2825–2830.

[ref19] Beebe K. R.; Pell R. J.; Seasholtz M. B. Chemometrics: A Practical Guide, 1998.

[ref20] Bro R. Multiway calibration. multilinear pls. J. Chemom. 1996, 10 (1), 47–61. 10.1002/(SICI)1099-128X(199601)10:1<47::AID-CEM400>3.0.CO;2-C.

[ref21] Jong S. d.; Wise B. M.; Ricker N. L. Canonical partial least squares and continuum power regression. J. Chemom. 2001, 15 (2), 85–100. 10.1002/1099-128X(200102)15:2<85::AID-CEM601>3.0.CO;2-9.

[ref22] Wold S.; Høy M.; Martens H.; Trygg J.; Westad F.; MacGregor J.; Wise B. M. The PLS model space revisited. J. Chemom. 2009, 23 (2), 67–68. 10.1002/cem.1171.

[ref23] Lackey H. E.; Sell R. L.; Nelson G. L.; Bryan T. A.; Lines A. M.; Bryan S. A. Practical guide to chemometric analysis of optical spectroscopic data. J. Chem. Educ. 2023, 100 (7), 2608–2626. 10.1021/acs.jchemed.2c01112.

[ref24] Lascola R.; O’Rourke P. E.; Kyser E. A. A piecewise local partial least squares (PLS) method for the quantitative analysis of plutonium nitrate solutions. Appl. Spectrosc. 2017, 71 (12), 2579–2594. 10.1177/0003702817734000. 28884597

[ref25] Ortiz M.; Sarabia L.; Herrero A.; Sánchez M.; Sanz M.; Rueda M.; Giménez D.; Meléndez M. Capability of detection of an analytical method evaluating false positive and false negative (ISO 11843) with partial least squares. Chemom. Intell. Lab. Syst. 2003, 69 (1–2), 21–33. 10.1016/S0169-7439(03)00110-2.

[ref26] Lee C.-Y.; Chang C.-L.; Wang Y.-N.; Fu L.-M. Microfluidic mixing: a review. Int. J. Mol. Sci. 2011, 12 (5), 3263–3287. 10.3390/ijms12053263. 21686184 PMC3116190

[ref27] Schwantes J.; Bryan S.; Orton C.; Levitskaia T. G.; Pratt S.; Fraga C.; Coble J. Advanced process monitoring safeguards technologies at Pacific Northwest National Laboratory. Proc. Chem. 2012, 7, 716–724. 10.1016/j.proche.2012.10.109.

[ref28] Andrews H. B.; Sadergaski L. R. Leveraging visible and near-infrared spectroelectrochemistry to calibrate a robust model for Vanadium (IV/V) in varying nitric acid and temperature levels. Talanta 2023, 259, 124554 10.1016/j.talanta.2023.124554. 37080075

[ref29] Angulo A.; Yang L.; Aydil E. S.; Modestino M. A. Machine learning enhanced spectroscopic analysis: towards autonomous chemical mixture characterization for rapid process optimization. Digital Discovery 2022, 1 (1), 35–44. 10.1039/D1DD00027F.

[ref30] Mishra P.; Passos D.; Marini F.; Xu J.; Amigo J. M.; Gowen A. A.; Jansen J. J.; Biancolillo A.; Roger J. M.; Rutledge D. N.; et al. Deep learning for near-infrared spectral data modelling: Hypes and benefits. TrAC Trends Anal. Chem. 2022, 157, 116804 10.1016/j.trac.2022.116804.

[ref31] Bocklitz T.; Schmitt M.; Popp J. Optical molecular spectroscopy in combination with artificial intelligence for process analytical technology. 2020.

[ref32] Dunn Z. D.; Bohman P.; Quinteros A.; Sauerborn B.; Milman F.; Patel M.; Kargupta R.; Wu S.; Hornshaw M.; Barrientos R.; et al. Automated online-sampling multidimensional liquid chromatography with feedback-control capability as a framework for real-time monitoring of mAb critical quality attributes in multiple bioreactors. Anal. Chem. 2023, 95 (49), 18130–18138. 10.1021/acs.analchem.3c03528. 38015205

[ref33] Sadergaski L. R.; Myhre K. G.; Delmau L. H. Multivariate chemometric methods and Vis-NIR spectrophotometry for monitoring plutonium-238 anion exchange column effluent in a radiochemical hot cell. Talanta Open 2022, 5, 100120 10.1016/j.talo.2022.100120.

[ref34] Arrigo L. M.; Jiang J.; Finch Z. S.; Bowen J. M.; Herman S. M.; Greenwood L. R.; Friese J. I.; Seiner B. N. Separation of lanthanide isotopes from mixed fission product samples. Separations 2021, 8 (7), 104 10.3390/separations8070104.

