# Supplementary material for: Automated Calibration for Rapid Optical Spectroscopy Sensor Development for Online Monitoring
Source: ACS Sens. 2024 Sep 19;9(11):6257–64. doi: 10.1021/acssensors.4c02211 (PMC11590107; doi:10.1021/acssensors.4c02211)
Supplement: Supplementary file 1 — se4c02211_si_001.pdf [file se4c02211_si_001.pdf]

Supporting Information

Automated Calibration for Rapid Optical Spectroscopy Sensor Development for Online Monitoring

Hunter B. Andrews[1,*], Luke R. Sadergaski[1]

[1]Radioisotope Science and Technology, Oak Ridge National Laboratory, 1 Bethel Valley Rd., Oak Ridge, Tennessee

Figures: 6

Tables: 2

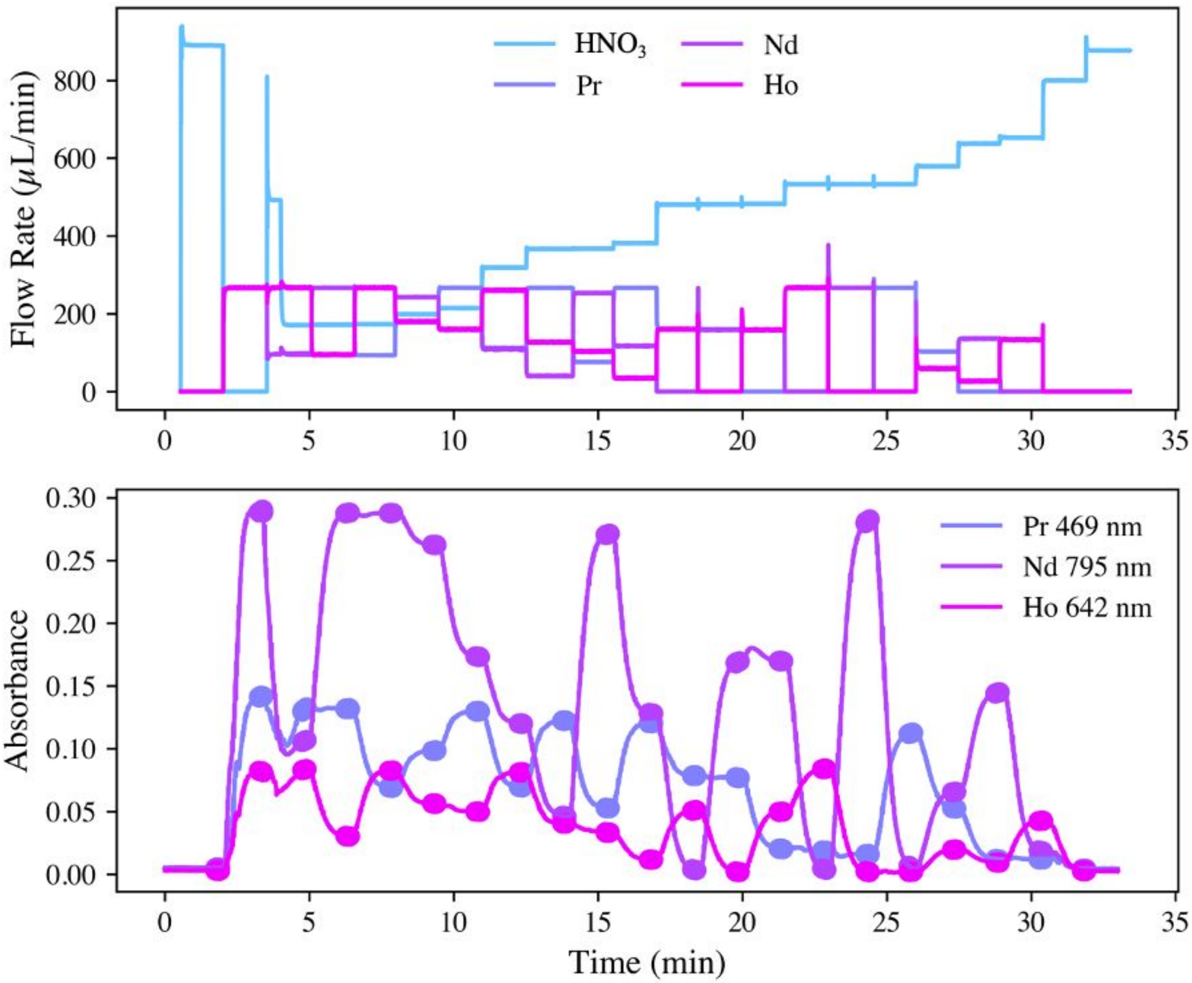

Figure S1. Comparison of (top) flow record and (bottom) univariate absorption response. Spectra selected for use in calibration are indicated by the markers in the bottom plot.

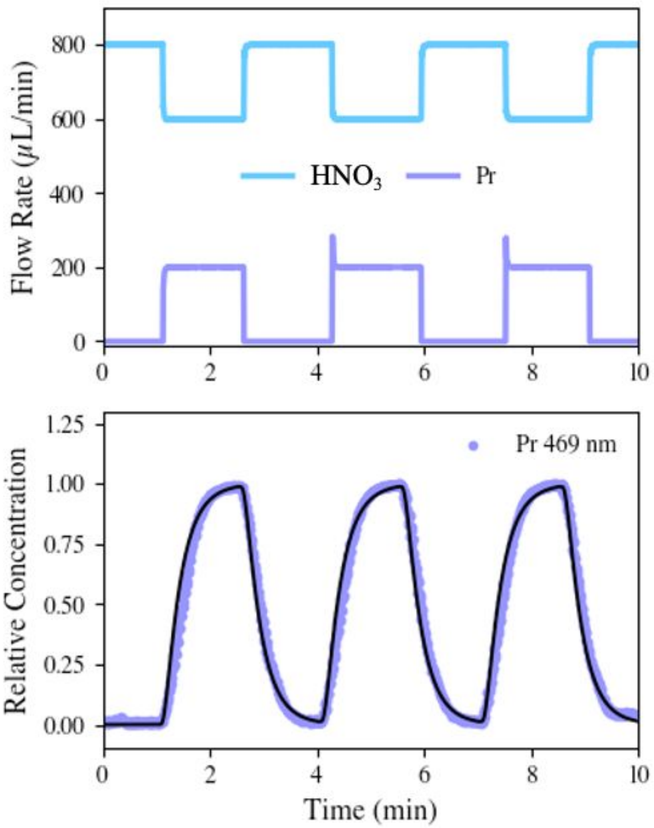

Figure S2. Demonstration of ATLAS mixing repeatability using a herringbone mixing chip. The expected concentration profile calculated using equation 2 is shown as the black line in the lower plot.

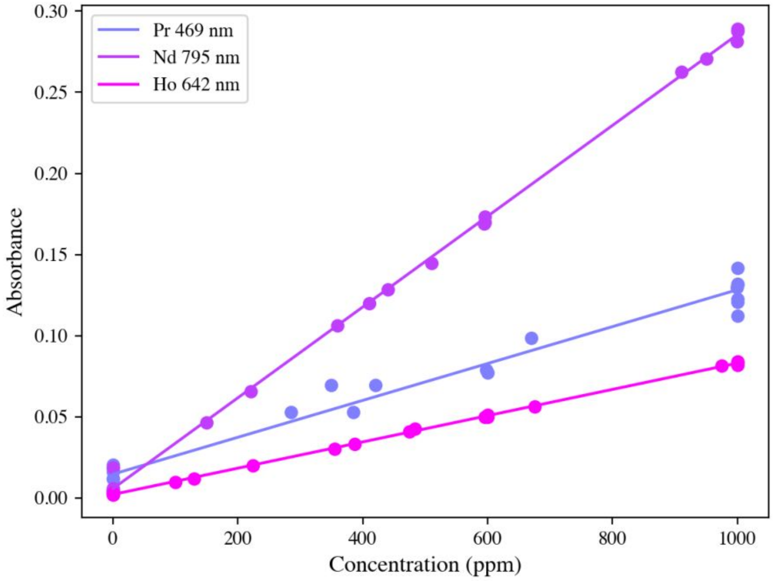

Figure S3. Pr, Nd, and Ho univariate response to concentration showing the variance in systems in which analyte peaks overlap with one another. The absorbance values were extracted from mixed samples. The R^2 values for these univariate fits were 0.974, 0.999, and 0.999, for Pr, Nd, and Ho, respectively.

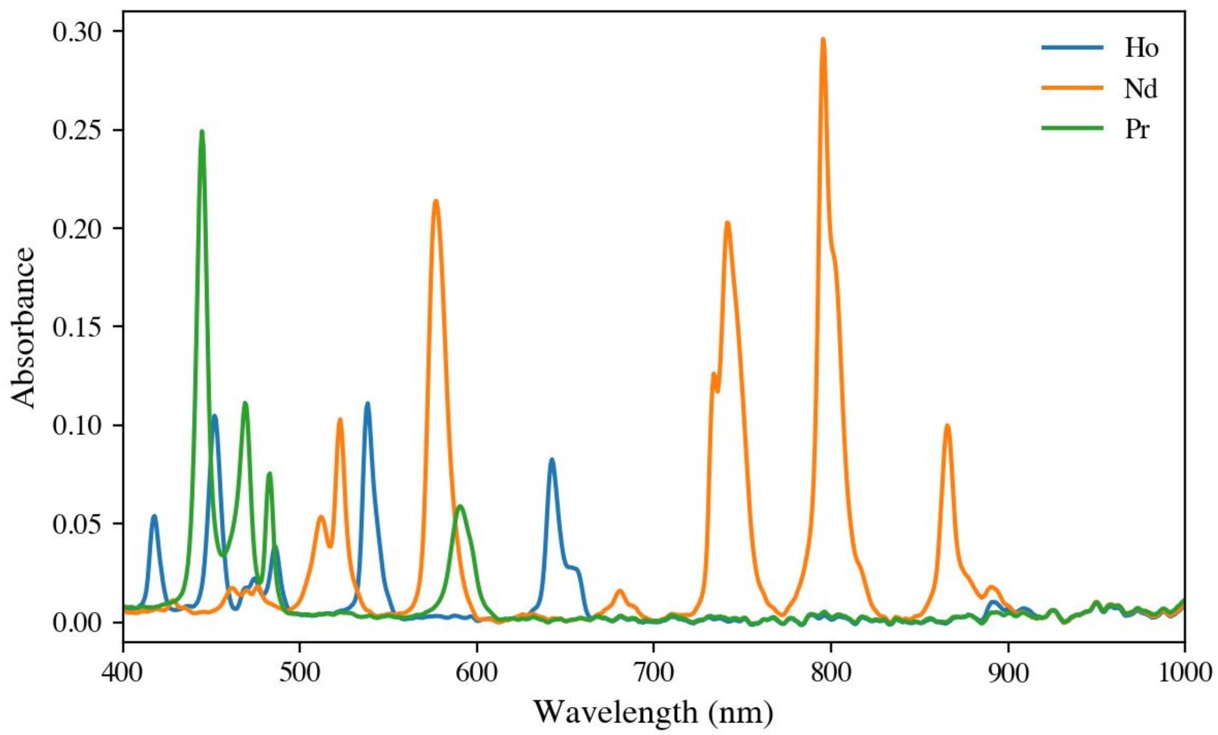

Figure S4. Absorbance profiles for individual Ho, Nd, and Pr systems at 1000 ppm, highlighting areas of interference.

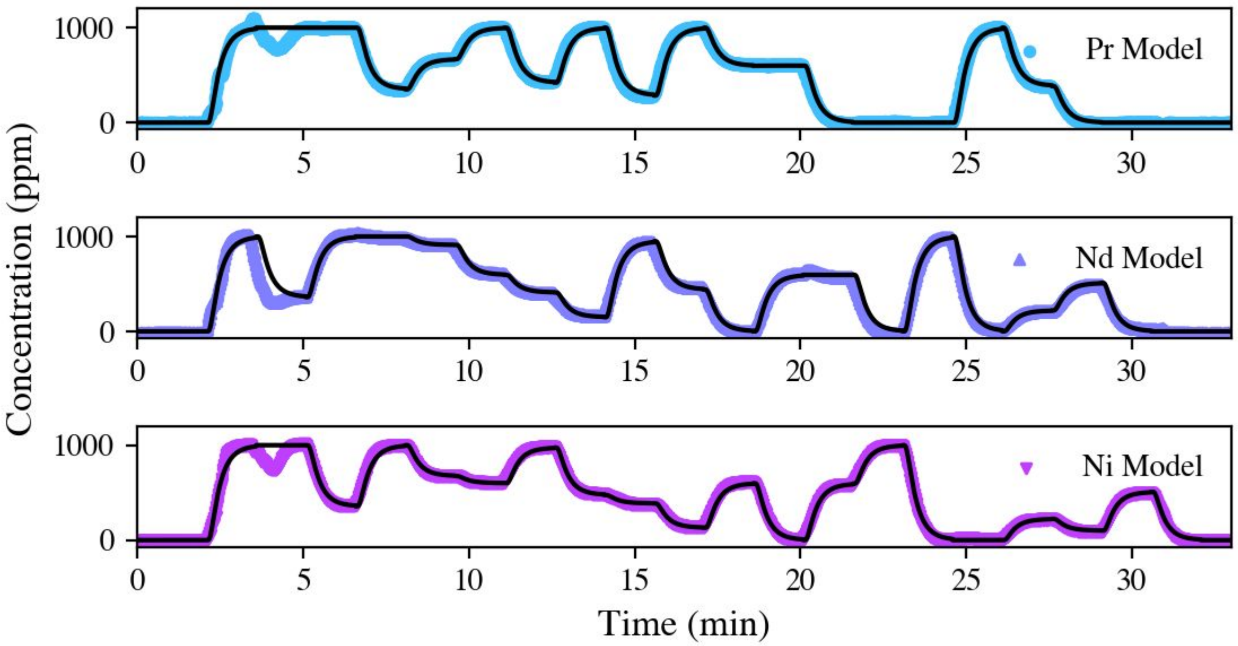

Figure S5. ATLAS PLSR model-predicted Pr, Nd, and Ni concentration profile compared with (black lines) expected profile.

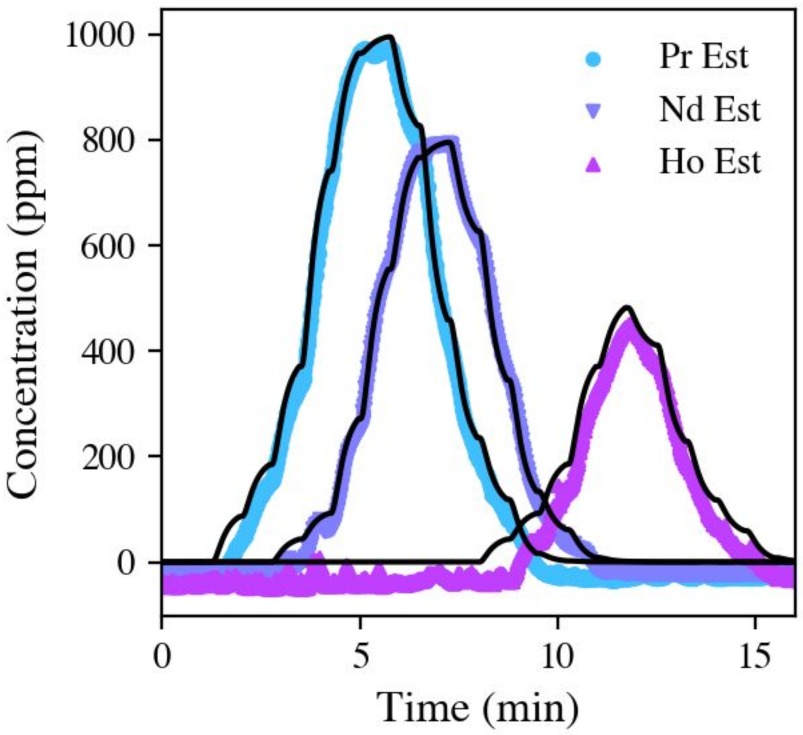

Figure S6. PLSR model concentration profile prediction of the simulated column run when simple baseline correction is used rather than derivative baseline correction. Notice the negative Ho predictions and fluctuations in the Ho profile prior to 8 min when no Ho was in the sample stream.

Table S1. Generic three-species D-optimal calibration plan.

Sample	Species 1	Species 2	Species 3	Space Type	Build Type
1	0.000	0.595	0.595	Plane	Model
2	1.000	0.595	0.600	Plane	Lack of Fit
3	0.000	0.510	0.100	Plane	Lack of Fit
4	0.385	0.220	0.224	Interior	Lack of Fit
5	0.000	0.000	0.000	Vertex	Model
6	0.350	1.000	1.000	Edge	Model
7	1.000	0.440	0.130	Plane	Lack of Fit
8	0.600	0.595	0.000	Plane	Model
9	0.598	0.000	0.600	Plane	Model
10	0.000	0.000	1.000	Vertex	Model
11	0.000	0.053	0.500	Plane	Lack of Fit
12	0.285	0.950	0.387	Interior	Lack of Fit
13	1.000	1.000	0.355	Edge	Model
14	0.669	0.910	0.675	Interior	Lack of Fit
15	0.420	0.410	0.975	Interior	Lack of Fit
16	1.000	0.150	0.475	Plane	Lack of Fit
17	1.000	1.000	1.000	Vertex	Lack of Fit
18	1.000	0.000	0.000	Vertex	Model
19	1.000	0.360	1.000	Edge	Model
20	0.000	1.000	0.000	Vertex	Model

Note: sample number does not indicate nor imply sample order for pumped tests. The space type column refers to where a concentration is located relative to the design space (*e.g.,* a vertex point is at the vertex of the cubic region of the design). The build type refers to whether the sample composition was included for the purpose of primary model development or checks to ensure a robust model (lack-of-fit).

Table S2. Pr, Nd, and Ho prediction metrics for ATLAS multivariate models when a first derivative Savitzky–Golay filter is applied.

Species	RMSEC (%)	RMSECV (%)	RMSEP(%)	R^2 Calibration	R^2 CV	R^2 Prediction	*LOD* (ppm)
Pr	1.2	1.4	8.4	0.9998	0.9992	0.9865	22
Nd	3.4	3.7	5.8	0.9981	0.9937	0.9928	59
Ho	0.91	1.2	6.0	0.9999	0.9993	0.9911	16

*PLSR models were built with LV = 3 (Pr), 2 (Nd), and 3 (Ho) and first derivative. *k*-fold CV was performed. RMSEP $n = 3211$.
